# The development and appraisal of a tool designed to find patients harmed by falsely labelled, falsified (counterfeit) medicines

**DOI:** 10.1186/s12913-017-2235-y

**Published:** 2017-06-20

**Authors:** Marija Anđelković, Einar Björnsson, Virgilio De Bono, Nenad Dikić, Katleen Devue, Daniel Ferlin, Miroslav Hanževački, Freyja Jónsdóttir, Mkrtich Shakaryan, Sabine Walser

**Affiliations:** 1 Sports Medicine Association of Serbia, 11000 Belgrade, Serbia; 2 Landspitali University Hospitali, Eiriksgata 5, 101, Reykjavík, Iceland; 3 Ordine dei Medici (Roma), Network of Family Doctors Rome, Rome, Italy; 4 VZW Stedelijk Ziekenhuis, 9300 Aalst, Belgium; 5 Istarski domovi zdravlja/Casa della salute del Istria, 52100 Pula, Croatia; 6 Obiteljska medicina, Zagreb, 10 000 Croatia; 7 Scientific Centre Drug and Medical Technology Expertise, 0051 Yerevan, Armenia; 8 Until March 2016: Department of Biological Standardisation, OMCL Network & HealthCare (DBO), European Directorate for the Quality of Medicines & HealthCare (EDQM), Council of Europe, CS 67081 Strasbourg, France

**Keywords:** Counterfeit medicines, Falsified medicines, Falsely labelled medicines (drugs, medicinal, products), Screening tool, Questionnaire

## Abstract

**Background:**

Falsely labelled, falsified (counterfeit) medicines (FFCm’s) are produced or distributed illegally and can harm patients. Although the occurrence of FFCm’s is increasing in Europe, harm is rarely reported. The European Directorate for the Quality of Medicines & Health-Care (EDQM) has therefore coordinated the development and validation of a screening tool.

**Methods:**

The tool consists of a questionnaire referring to a watch-list of FFCm’s identified in Europe, including symptoms of their use and individual risk factors, and a scoring form. To refine the questionnaire and reference method, a pilot-study was performed in 105 self-reported users of watch-list medicines. Subsequently, the tool was validated under “real-life conditions” in 371 patients in 5 ambulatory and in-patient care sites (“sub-studies”). The physicians participating in the study scored the patients and classified their risk of harm as “unlikely” or “probable” (cut-off level: presence of ≥2 of 5 risk factors). They assessed all medical records retrospectively (independent reference method) to validate the risk classification and documented their perception of the tool’s value.

**Results:**

In 3 ambulatory care sites (180 patients), the tool correctly classified 5 patients as harmed by FFCm’s. The positive and negative likelihood ratios (LR+/LR-) and the discrimination power were calculated for two cut-off levels:

a) 1 site (50 patients): presence of two risk factors (at 10% estimated health care system contamination with FFCm’s): LR + 4.9/LR-0, post-test probability: 35%;

b) 2 sites (130 patients): presence of three risk factors (at 5% estimated prevalence of use of non-prescribed medicines (FFCm’s) by certain risk groups): LR + 9.7/LR-0, post-test probability: 33%.

In 2 in-patient care sites (191 patients), no patient was confirmed as harmed by FFCm’s.

The physicians perceived the tool as valuable for finding harm, and as an information source regarding risk factors.

**Conclusions:**

This “decision aid” is a systematic tool which helps find in medical practice patients harmed by FFCm’s. This study supports its value in ambulatory care in regions with health care system contamination and in certain risk groups.

The establishment of systematic communication between authorities and the medical community concerning FFCm’s, current patterns of use and case reports may sustain positive public health impacts.

**Electronic supplementary material:**

The online version of this article (doi:10.1186/s12913-017-2235-y) contains supplementary material, which is available to authorized users.

## Background

Falsely labelled, falsified (counterfeit) medicines (FFCm’s) pose a health risk: they often do not contain the correct ingredients (or those labelled), may have been trafficked by criminals and may have been contaminated with toxic substances during non-regulated production and distribution.

The occurrence of FFCm’s is increasing everywhere, including in Europe [1–8]. Although attempts at removal from the market remain incomplete [9, 10], health damage in Europe is not visible in health statistics: this may be explained in part by a lack of specific vigilance and awareness as well as under-reporting.

The early detection of signs and symptoms of health damage caused by FFCm’s can support decision- making on treatment, but is at present hampered by the unavailability of validated screening tools.

A screening tool needs to consider the main factors influencing the extent of health damage caused by FFCm’s in an individual, such as the types and quality of defects of circulating FFCm’s and/or patterns of their use.

## Methods

### Study aim

The aim was firstly to develop and validate a tool which can support physicians to find patients harmed by FFCm’s and secondly to assess the added value of the tool to physicians.

#### Study design

The study was observational. It did not require diagnostic tests or therapies in addition to those which would have been carried out by the health care site to treat the patient. The design took the following circumstances into account under which the tool would be used: health care system contamination with FFCm’s and/or purchases of medicines/health care products by individuals from uncontrolled outlets.

### Development of the tool

A preliminary concept for an “anamnesis” form for clinical use was produced by experts in the framework of a specific work program coordinated by the EDQM, Council of Europe.^1^ The concept was mentioned in a preliminary report [11]. Based on this concept, we further developed the tool and designed the study approach, regularly consulting with the above experts as well as with those from the national official medicines control laboratories (OMCL). These laboratories carry out market surveillance programmes in Europe, and can thus be used as a reference for the quality of medicines including issues with substandard and FFCm’s [12].^2^

The tool was designed to complement usual diagnosis and treatment-planning processes during consultation: it consisted of 1) a questionnaire, 2) a watch-list of FFCm’s identified in Europe, relevant patterns of their use (“lifestyles”) and clinical signs and symptoms and 3) a scoring form (See “Tool: Questionnaire and scoring form” in Additional file 1. The watch-list is available upon request from the EDQM contact person. See “Data and Materials”).

The following 5 domains are included in the questionnaire:

- health complaints;
- medical history;
- use of medicines;
- use of health care products;
- life-styles relevant for exposure to FFCm’s.

The scoring form comprised these 5 domains which contributed equally (20 scores each) to the total score of 100. The information provided by the patient on each domain was used by the physician to judge whether a risk factor of exposure to FFCm’s associated to this domain was present or absent and to assign the scores accordingly.

We selected the medicine classes for inclusion in the watch-list in line with the findings of the preliminary report [11] which had identified 7 therapeutic classes frequently seized in Europe as FFC. Amongst them were medicines with psycho-analeptic, antibiotic, anabolic properties, as well as medicines to treat cardiovascular diseases, erectile dysfunction, obesity and various health care products.

Three experts (an endocrinologist, a psychiatrist, and a pharmacist) prepared the watch-list section „clinical signs“ and the life-style questions included in the questionnaire. Information regarding the signs associated with the use of 56 individual medicines (brands) belonging to the above 7 therapeutic classes was sourced from literature.

Those 56 individual medicines had been identified as FFC by the OMCLs. The types and extent of the quality defects had been confirmed through analytical methods.

The life-style questions (25 in total) addressed behaviours and attitudes of patients associated with a risk of using medicines and health care products to alter body appearance or performance. The selection of these questions was based on data regarding patterns of their use [5] and on the experts’ own clinical experiences and were phrased in a non-blaming and non-embarrassing style.

All physicians participating in the sub-studies contributed to the development of the tool and the study processes before the beginning of the study in oral and written communication. (Selection of the participating physicians, see “Setting and participants”).

The tool, the reference method and the study processes were pre-tested in a pilot-study which was carried out in self-reported users of watch-list-medicines in an ambulatory care study site (Belgrade, Serbia).

### Reference method (validation of the tool)

The retrospective assessment of the medical records of all patients including all diagnostic tests performed after the initial consultation, in particular biomarkers and clinical signs or symptoms, response to medical treatment and progress of disease, was used as the reference method (see Materials and Processes, Step 3). In order to compile possible signals (relevant (bio)markers, other clinical findings and the progress of disease) for the retrospective assessment, a pilot-study was carried out in a sports clinic (Belgrade, Serbia) in 105 athletes. They reported use of most classes of the watch-list-medicines: anabolic agents, anti-obesity preparations, sex hormones and modulators of the genital system including medicines for erectile dysfunction, diuretics and a multitude of healthcare products, and presented health complaints. Nearly all medicines (90%) were purchased from poorly controlled or uncontrolled outlets and were possibly falsely labelled, falsified (counterfeit). The tool was used according to the study processes and the use and origin of the medicines, relevant (bio)markers, other clinical findings and the progress of disease was documented and shared with the participating physicians.

### Setting and participants

The study-setting comprised 1 pilot-study and 5 sub-studies in ambulatory and in-patient care sites in 6 different countries in Europe (Table 1). Experts (1) were consulted to select those classes of medicines for the validation study which were relevant in the respective country from the perspective of public health protection. These experts also identified the health care sites and the participating physicians. Each ambulatory care site was expected to test the tool in 30 patients using medicines belonging to the selected class(es) during the 3-months study period (2013–2014). Each in-patient site was expected to test the tool in 100 patients.

**Table 1:** Health care settings, exclusion criteria, patient characteristics, selected medicines classes

Health care setting	Exclusion criteria: All sub-studies: female patients as regards erectile dysfunction medicines(G04BE)	Number: enrolled (evaluated)	Sex (m/f)	Age (*Median, Interquartile range Q1-Q3*): all patients; (m/f)	Medicines classes (WHO ATC)
*Ambulatory* Yerevan (Armenia)	Patients of all age groups with mild to moderate infections	50	24/26	33 (*20.5–45.5*) (34 (*19*–*49*)/ /31 *(19.5–42.5)*	Antibiotics (J01)
*Ambulatory* Zagreb & Pula (Croatia)	Patients <14, > 65 y;	71 (70)^a^	34/36	47 (*35*–*59*) (48 (*37.7–58.5*)/ 43.5 (*30.5–56.5)*	Anti-obesity Preparations, Excl. Diet Products (A08), Anabolic Agents for Systemic Use (A14), Diuretics (C03), Sex Hormones and Modulators of the Genital System (G03), Urologicals (G04), Psycho-analeptics (N06)
*Ambulatory* Rome (Italy)	Patients <14, > 65 y	60 (60)	60 m	m: 32 (*25.4–38.6*)	Anabolic Agents for Systemic Use, Diuretics, Sex Hormones and Modulators of the Genital System, Urologicals
Pilot-study *Ambulatory* Sports out-patient clinic Belgrade (Serbia)	Patients <14, > 65 y;	105 (105)	79/26	33 (*27*–*39*) (33 (*26.5–39.5*)/ 33 (*28*–*38*))	Anti-obesity Preparations, anabolic agents for systemic use, Diuretics, Sex Hormones and Modulators of the Genital System
*In-patient* Aalst (Belgium)	Patients <14, > 65 y;	42 42	23/19	42 (*27.4–56.7*) (45 (*27.3–53.8)* 42 (*30*–*54*)	Anti-obesity Preparations, Anabolic Agents for Systemic Use, Diuretics, Sex Hormones and Modulators of the Genital System, Urologicals, Psycho-analeptics
*In-patient* Reykjavík (Iceland)	Patients <14, > 65 y;	149 (149)	51/98	47 *(32.5– 61.5)* (46 *(31.5–60.5)*/ 48 *(34–62)*	Anti-obesity Preparations

Legend: WHO ATC: Anatomical Therapeutic Chemical Classification of Medicinal Products. Source: World Health Organization (WHO). (http://www.whocc.no/atc_ddd_index)

^a^One (1) patient was excluded due to incomplete documentation

In general, patients below the age of 14 years and those aged above 65 years were excluded (Table 1), with the exception of the sub-study in Yerevan: this study focused on antibiotic treatment and enrolled patients with mild to moderate infections, irrespective of their age.

### Materials and processes

Electronic files of the tool were provided to the participating physicians, either complete or limited to the classes of medicines selected for the respective sub-study. The participating physicians were trained during phone conferences, with respect to the study processes, sensitive questioning and avoidance of pre-selection of patients by personal judgement. A meeting was held for the participating physicians after the data lock point to discuss practical details of the retrospective assessment of patients’ medical records (see Statistical analysis).

The study processes comprised 3 steps:

Step 1: The physician included the questionnaire in the questioning of the patient at the first consultation and scored the information using the watch-list. The physician established whether harm caused by FFCm’s was “probable” requiring the presence of at least 2 of 5 risk indicators (corresponding to ≥ 40 scores), or “unlikely”.
Step 2: The therapy and further diagnostic interventions were carried out in line with the site’s usual protocols.
Step 3: After the patient had been discharged from in-patient care or after alleviation of symptoms (ambulatory care), the physician assessed the patient’s medical record, regarding
  all diagnostic tests performed after the initial consultation, in particular biomarkers and clinical signs or symptoms,
  response to medical treatment,
  progress of disease.

In the sub-study focusing on FFC antibiotics, consecutive antibiograms were assessed in accordance with medical guidelines.

The physician documented on the scoring form whether the above findings maintained the initial risk status („true positive” or “true negative”) or provided grounds for rejection (“false positive” or “false negative”).

### Statistical analysis

Standard descriptive statistics were used to analyse count variables and continuous variables (Tables 1 and 2).

**Table 2:** Discrimination power of the tool

**Health care setting/Medicines**	**True positives** ^**a**^ **(n)**	**False positives** ^**b**^ **(n)**	**Cut-off level (scores) of 100:** Sensitivity/Specificity (%) (Confidence intervals^c^: Lower 95% CL; Upper 95% CL); Positive/Negative^d^ Likelihood ratios (LR+/LR-)			
*Ambulatory* Yerevan (Armenia)/Antibiotics	1	10	**≥40:**			
			Sensitivity	100	(2.5	100)
			Specificity	79.95	(65.66	89.76)
			**LR+**	**4.9**		
			LR-	0		
	0	2	≥60			
			Sensitivity	NaN	(0	100)
			Specificity	96	(86.29	99.51)
			LR+	N/A		
			LR-	NaN		
*Ambulatory* Zagreb & Pula (Croatia)/Anti-obesity Preparations, Anabolic Agents for Systemic use, Diuretics, Sex Hormones and Modulators of the Genital System, Urologicals, Psycho-analeptics	1	20	≥40			
			Sensitivity	100	(2.5	100)
			Specificity	71.01	(58.84	81.31)
			LR+	3.5		
			LR-	0		
	1	13	**≥60**			
			Sensitivity	100	(2.5	100)
			Specificity	81.16	(69.94	89.57)
			**LR+**	**5.3**		
			LR-	0		
*Ambulatory* Rome (Italy)/ Anabolic Agents for Systemic use, Diuretics, Sex Hormones and Modulators of the Genital System, Urologicals	3	0	**≥40; ≥60**			
			Sensitivity	100	(29.24	100)
			Specificity	100	(93.73	100)
			LR+	-		
			LR-	0		
*All ambulatory sub-studies* Yerevan (Armenia), Zagreb & Pula (Croatia), Rome (Italy)						
	5	30	≥40			
			Sensitivity	100	(47.82	100)
			Specificity	82.86	(76.44	88.12)
			LR+	5.8		
			LR-	0		
*Ambulatory sub-studies* Yerevan (Armenia), Zagreb & Pula (Croatia), Rome (Italy)						
			≥40			
			Sensitivity	100	(39.76	100)
			Specificity	84.13	(76.56	90.03)
			LR+	6.3		
			LR-	0		
			**≥60**			
			Sensitivity	100	(39.76	100)
			Specificity	89.68	(83	94.39)
			LR+	9.7		
			LR-	0		
*In-patient* Aalst (Belgium)/Anti-obesity Preparations, Anabolic Agents for Systemic Use, Diuretics, Sex Hormones and Modulators of the Genital System; Urologicals, Psychoanaleptics	0	33	≥40			
			Sensitivity		0	100
			Specificity	21.43	(10.3	36.81)
			LR+	N/A		
			LR-			
	0	11	**≥60**			
			Sensitivity	0	100	
			Specificity	73.81	(57.96	86.14)
			LR+	N/A		
			LR			
*In-patient* Reykjavík (Iceland)/ Anti-obesity Preparations	0	13	≥40			
			Sensitivity	0	100	
			Specificity	92.52	(87.01	96.21)
			LR+	N/A		
			LR-			
	0	5	**≥60**			
			Sensitivity	0	100	
			Specificity	96.64	(92.34	98.9)
			LR+	N/A		
			LR-			

Legend

The cut-off level which classified correctly the cases of harm at a minimum of false classifications is highlighted in **bold** letters

^a^“True positive”: The initial classification of the patient by the tool as “probably being at risk of health damage caused by FFC medicines” was maintained after the retrospective assessment of the medical record (“reference method”)

^b^“False positive”: The initial classification of the patient by the tool as probably being at risk of health damage caused by FFC medicines” was rejected after the retrospective assessment of the medical records (“reference method”)

^c^The sensitivity and specificity of the tool were expressed as “exact two-sided 95% confidence intervals” according to the method of Clopper and Pearson for the presence of 2 and 3 of 5 risk indicators

^d^Positive/negative likelihood ratios: the ability of a test to discriminate between people likely to have a disorder and those less likely to have a disorder is determined by the test’s likelihood ratio (LR)

The ratio of true positives to false positives (LR+) is the likelihood ratio for a positive test result being correct

The ratio of false negatives to true negatives (LR-) is the likelihood ratio for a negative test result being correct

The results from the pilot-study in patients self-reported users of watch-list medicines (Belgrade, Serbia) were not included in the statistical analysis of the discrimination power of the tool, because the patients’ risk status was known. (See Methods: Reference method (validation of the tool).

Correctly and falsely classified patients (absolute frequencies) were stratified into ambulatory and in-patient care sites and for each individual sub-study. The tool’s power to discriminate between patients harmed by FFCm’s and those who were not harmed was evaluated with respect to “sensitivity” and “specificity”, likelihood ratios, and predictive values (see legend Table 2). The sensitivity and specificity of the tool was expressed as “exact two-sided 95% confidence intervals” according to the method of Clopper and Pearson [13–15] (Table 2) for 2 and for 3 of 5 risk indicators in order to establish a discrimination threshold (“cut-off level”) which correctly classifies cases of harm at a minimum of false classifications.^3^ The post-test probability was estimated using Fagan’s Nomogram for Bayes’s Theorem [16] adapted for interpreting diagnostic results, based on estimates of prevalence of health care system contamination with FFCm’s and of users of non-prescribed medicines from uncontrolled outlets, often FFC. The implications of setting a cut-off level (≥40 or ≥ 60 of 100 scores corresponding to the presence of 2 or 3 risk factors, respectively) on the discrimination power of the tool are addressed in the discussion.

Added value (convenience of use) of the tool to physicians: The participating physicians provided their perception via a questionnaire with open-ended questions upon completion of the sub-study. Their views were analysed in a qualitative manner by clustering them according to topic and emphasis (See Table 3).

**Table 3:** Added value (convenience of use) of the tool

Health care setting	Participating physicians’ views
*Ambulatory* Yerevan (Armenia)	“…The approach proved suitable for use in case of unavoidable exposure to FFCm’s contaminating the legal chain for example essential medicines (antibiotics)… and deliberate purchases from uncontrolled sources …”.
*Ambulatory* Zagreb& Pula (Croatia)	“…Systematic approach for the identification of risk factors… it justifies the consideration of alternative diagnosis; opportunity for counselling of patient (preventative role).... …additional anamnestic questions increased the duration of the consultation on average 6. 2 min (range: 3. 2- 11.4 min). However this time should not be considered a mere prolongation of the consultation: it helps establish the suspicion of possible “poisoning” which by itself has to be clarified thoroughly. Physicians have an inherent and professional interest to understand the nature of the observed symptom. Our questionnaire only helps do that. ”
*Ambulatory* Rome (Italy)	“…The decision aid is useful to find patients at risk, combining known risk factors with the medical history of individual patients; but also offering alternatives for possible diagnosis (“differential diagnosis options”) to document and justify further diagnostic interventions.... The present observational study, though small, showed the existence of unsafe use of medicines (like phosphor-diesterase inhibitors), consumption of psychoactive substances and substances with a doping effect, as well as the indiscriminate use of dietary supplements which possibly cause disabling symptoms and a medical referral. …We can say that the protocol adopted here is potentially useful to define not only the characteristics of subjects potentially at risk for the use and abuse of medicines in the differential diagnosis of a condition possibly iatrogenic but also useful to identify with greater specificity the need for further investigation diagnostics. The use of the decision aid requires 20–30 min. It is most important is that the physician does not question the patient in a ”sterile manner“ but tries to understand personal motivation (problems) through direct and indirect questions ….”.
Pilot-study *Ambulatory* Belgrade (Serbia)	“…Health care settings with easy access (primary care) and mid to long- term trusted relationship are suitable…” …Welcome the decision aid: this is, for the first time, a tool set up specifically for doctors. The primary care sector, such as family doctors, who are easy to access and have usually a longer-term, trusted relation with the patients, is well positioned to find patients at high risk of health damage by medicines produced and distributed outside the legal chain such as counterfeit (falsified) medicinal products….. …In consultations, time is limited: risk status output needs to be integrated into patients’ questioning (anamnesis)…”.
*In-patient* Aalst (Belgium)	“….The decision aid gives an important reminder to the doctor – to take into account in treatment decisions and risk prevention, adverse reactions caused by FFC products; it could be a useful complement …. …Manual scoring (*used in this observational study setting*) is impractical in real-life situation…..”
*In-patient* Reykjavík (Iceland)	“…There is no doubt that the topic of this project is very important. In my opinion it is more important that doctors always bear this in mind and include questions on the use of alternative medicines and/or counterfeit medicines. The consultation tool does not seem to be cost effective for use in unselected patients presenting in a general practice or in a gastroenterology clinic. …Biomarkers (*of health damage caused by FFC products*) are needed …”.

Legend: The participating physicians provided their views (perception) of the added value and the convenience of use of the tool via a questionnaire with open-ended questions

## Results

### Discrimination power

Sensitivity, specificity, likelihood ratios of all sub-studies (in the presence of 2 or 3 of 5 risk factors) are listed in Table 2.

### Ambulatory care setting sub-studies

#### Yerevan (Armenia)

Fifty patients with mild to moderate infection of different aetiology presented in the general practitioner’s office. One of the 11 cases scoring above 40 scores, corresponding to the presence of 2 of 5 risk factors, was maintained as true positive: urinary infection by *Staphylococcus aureus* had been confirmed through an antibiogram. Rocephin® (ceftriaxone) was administered for 7 days (1 g/day). A subsequent antibiogram showed continued presence of the microorganisms and sensitivity to ceftriaxone. Second line treatment, rovamycine, was successful. The third microbiological analysis demonstrated that *Staphylococcus aureus* was no longer present.

Additionally, the tool helped find 3 patients who reported use of “biologically active supplements” to improve their performance in sports. As supplements advertised as “biologically active” are known to contain hormones for muscle growth, patients could be counselled during the consultation about the health risk associated with the use of those products.

#### Zagreb and Pula (Croatia)

Seventy one patients attended two offices of the same general practitioners’ network for non-serious ailments and chronic diseases. One of the 21 cases scoring above 40 scores corresponding to the presence of 2 of 5 risk factors, was maintained as true positive: the patient presented with increased levels of hepatic enzymes for more than 3 days, a sign of hepatic disorder, and was therefore referred to the hospital. At the initial consultation, the patient had reported the use of a dietary supplement. The hospital’s discharge report confirmed hepatic disorder due to the use of a product containing the anabolic agent oxandrolone.

#### Rome (Italy)

Sixty patients with non-serious health complaints presented at the offices of a general practitioners’ network. 15% of the patients reported practising sport, one professionally, the others for leisure.

Three patients with cardiovascular symptoms (elevated blood pressure, wheezing, dizziness, and arrhythmia) scored above 40 scores corresponding to the presence of 2 of 5 risk factors. These 3 patients denied practising sport. One of them reported using sildenafil, a medicine to treat erectile dysfunction. At the initial consultation, none of them reported use of other medicines, vitamins and amino acids (health care products). Further medical examination established disease (cardiopathies) and altered hepatic enzyme profile and testosteronaemia.

In the course of subsequent treatment it was confirmed that the patients had used the anabolic agents for systemic use: nandrolone decanoate, testosterone enanthate, mesterolone, boldenone undecyclenate, trenbolone hexahydro-benzylcarbonate, testosterone propionate and stanazolol.

In the whole patient group, the use of vitamins and dietary supplements (protein-amino acid vitamin mix, creatin mix, “Thermoburner Animals stack mix”) was common: 25 patients reported use of supplements upon the recommendation of “…fitness-trainers, friends, family members or physicians…”.

### In-patient setting sub-studies

#### Aalst (Belgium)

Fourty two patients admitted to the emergency unit presented with pain due to traumatic injury or internal or psychiatric diseases. 33 patients scoring above 40 scores corresponding to the presence of 2 of 5 risk factors, were not maintained (they were falsely classified as positive). They did not report use of medicines or health care products included in the watch-list.

The assessment of the medical records identified severe traumatic injury, internal or psychiatric diseases as causal of the symptoms.

#### Reykjavík (Iceland)

One hundred fourty nine patients presented with gastroenterological symptoms, pain, and mood alterations (psychiatric symptoms) at the internal medicine department. 13 patients scoring above the cut-off level of 40 scores corresponding to the presence of 2 of 5 risk factors, were classified retrospectively as false positive: Among these patients, were 4 patients who had reported the use of watch-list-medicines and healthcare products: one patient had used a product stimulating the thyroid gland with no relevant medical history, and the other 3 patients had used herbal products. The retrospective assessment of the medical records of those 4 patients suggested anaemia in 2 patients, Wilson’s (hepatic) disease in 1 patient, and gastro-intestinal tract disorder in 1 patient as causal of their health complaints.

The frequencies of health complaints, usage of medicines and health care products before consultation, and medical history are included in (Additional file 2: Table S1): In summary, patients’ medical history and health complaints were consistent with the reported medicines’ usage.

#### Added value (convenience of use)

The participating physicians from all sub-studies, including the pilot-study, gave their views on the “added value” and “convenience of use” of the tool (Table 3). In general, the physicians perceived the tool as a valuable element of patient questioning during consultations, as there was consensus that the prevention of health damage caused by FFCm’s is important.

One physician (Zagreb, Croatia) estimated that the time needed to complete the questionnaire during consultations took on average about 6 min (from 3. 2 to 11. 4 min).

The physicians considered the ambulatory care setting as “well-placed” to find patients harmed by FFCm’s and to carry out effective patient counselling, particularly, if there was a long-standing and trusted patient-doctor relationship. They were of the opinion that patients would often hesitate to answer straight-forward questions regarding the use and origin of medicines and health care products for life-style purposes (weight-loss, beauty, sports) or sexual performance: therefore, sensitive life-style questioning, as supported by the tool, is most suitable for obtaining information. The physicians participating in the in-patient care sub-studies considered the tool as not being cost-effective in a sample of “non-selected” patients, in particular if the occurrence of FFCm’s was low. They recognised, however, the practical value of the tool as an “aide memoire” about types and clinical symptoms caused by FFCm’s.

All physicians considered manual scoring as required by this preliminary version of the tool as too time-consuming for medical practice.

## Discussion

The discussion of the implications of the study results with regard to the study aims requires a rather detailed analysis.

### Discrimination power of the tool

Ambulatory care: One sub-study focused on essential medicines (antibiotics) in Yerevan, Armenia, a region where the estimated level of contamination of the health care system with FFC medicines is 10-20% [17]. The incidence of counterfeit Rocephin® in Armenia before and during the study period had been confirmed by the competent authorities. It is therefore plausible that one patient (out of 50 patients using more than one medicinal product at a time) was classified correctly as “probably” harmed by a FFC antibiotic. This case was identified at a cut-off level of ≥ 40 scores corresponding to the presence of 2 of 5 risk factors.

Although the LR+ 4.9/LR- 0 generates only a moderate effectiveness in diagnosing if a patient is harmed by FFCm’s, it moves the post-test probability to about 35 – 60% for an estimated level of health care system contamination of 10 – 20% with FFCm’s (prevalence). Increasing the cut-off level to 3 of 5 risk factors (≥60 scores) would have missed the case. This may in similar cases put patients at risk of undertreatment of their infection and contribute to the development of bacterial resistance. Furthermore, the early detection of the FFC antibiotic and replacement by a genuine medicine could, in similar cases, render unnecessary a switch to second line antibiotics which are often more toxic.

In contrast to low-income countries with wide-spread health care system contamination with FFCm’s exposing a considerable proportion of the population to FFCm’s, the contamination of the health care system is very low (<1%) in Middle-Europe. However, a considerable number of individuals are purchasing medicines without the required prescription, e.g. for erectile dysfunction or anabolic agents, or health care products for life-style purposes (slimming and beauty) from non-regulated outlets, such as the internet. This could be risky as 50% of the medicines distributed via internet sites which do not reveal their identity are probably falsified [17]. The two sub-studies in Rome (Italy) and Zagreb and Pula (Croatia) focused on the risk of health damage caused by FFC products purchased from uncontrolled outlets in the setting of the offices of a general practitioners’ network.

In these 2 sub-studies, four out of 130 patients (3%) were correctly classified as probably harmed by FFC medicines or products. This is plausible in the light of an EU Commission study (2002) [18] which revealed that, on average, 5% of fitness centre clients, including school children and students, in the four member states concerned (Belgium, Germany, Italy, Portugal) reported the regular use of anabolic steroids, diuretics, stimulants, food supplements and anti-oestrogens. A cut-off level of ≥ 40 scores corresponding to the presence of 2 of 5 risk factors classifies correctly the cases of harm but results in elevated numbers of false classifications. Moving the cut-off level from ≥ 40 scores to ≥ 60 scores in the above 2 sub-studies reduces the number of false positives from 20 to 13 and maintains the number of 4 correctly classified cases. Therefore, we think that a cut-off level of ≥ 60 scores, corresponding to the presence of 3 risk factors of 5 risk factors, including the impact of life-style choices, is more proportionate to a scenario where FFCm’s are purchased by individuals via un-controlled outlets and can augment the usefulness of the tool in this setting. The resulting LR+ 9.7/LR- 0 can generate a moderate to larger effectiveness in diagnosing if a patient is harmed by FFCm’s or health products, moving the post-test probability to about 33% in this specific population group at risk (5% prevalence of use of non-prescribed medicines in fitness club clients).

The relevance to public health is illustrated by the following data: In Italy, 32% of all fitness centre clients are aged between 18–25 years [19], the age group which most intensely practises body-building. In 2014, 6 074 293 persons (9, 8% of the population) [20] were aged between 15–24 years.

In-patient care: In one hundred and ninety one patients, no patient was classified correctly as harmed by watch-list-medicines and health care products. We assume that in this sample of patients, who suffered either from traumatic injury and severe internal or psychiatric diseases, patients harmed by FFCm’s were under-represented. This may particularly apply to the patients enrolled in the sub-study in Reykjavík, which focused on health damage caused by one medicine class, FFC anti-obesity preparations. Given the small population size of Iceland and the relatively short study period, it is probable that patients harmed by FFC slimming products were not represented.

### Added value (convenience of use) of the tool

Our study aimed at a “proof of principle” of the tool and did not carry out a cost/benefit calculation for the use of the tool.

The participating physicians considered the use of the questionnaire as time-effective support to patient questioning during consultation. In particular, they welcomed the development of the specific watch-list combining the main factors that influence the extent of health damage caused by FFCm’s in an individual patient presenting clinical signs associated with the use of FFCm’s. The tool provides for sensitive and empathic questioning which is particularly important when addressing topics such as erectile dysfunction, slimming and use of performance enhancing products in sports. Experience shows that many patients will not speak openly when asked directly by a doctor about the products they took and where they had purchased them. The study results suggest that the tool can support the physician in carrying out a comprehensive patient questioning during consultation, including risk factors of harm caused by FFCm’s.

In order to solve the issues with time-consuming manual scoring and searching the watch-list raised by the physicians, common table calculation software could be used as a first step to digitalise the tool.

Systematic and continuous communication between the health authorities and the medical and health professionals’ community concerning new types and quality defects of FFCm’s and illegal healthcare products, patterns of their use and clinical signs and symptoms of associated health damage are needed to foster the sustainable and effective implementation of the tool in practice. Regular systematic updates and follow-up with the medical community are important to build and maintain a “pool” of expertise and motivation. Both aspects are part of the development concept and the design of the tool which permits easy modification, as necessary. Further research is recommended on the digitalisation of the tool, biomarkers and possible uses of the tool to promote the awareness of physicians of FFCm’s.

### Study strengths and limitations

A strict procedure was followed in order to minimise a possible “expectation bias” which could have distorted the results of the retrospective assessment (reference method). The physician, coordinator of the pilot-study (Belgrade, Serbia), reviewed the retrospective assessment of all medical records and the patient data after the completion of the whole study and compiled the study data. This physician was not involved in scoring the patients of the sub-studies or in the subsequent treatment.

It is a study limitation that samples of the medicines were not available for analysis and no technically sophisticated diagnostic analyses were carried out. Although such methods could be considered a “gold standard”, validated chemical-analytical methods are not always available and their use would limit the wide-spread use of the tool under “real-life” conditions.

In line with published data [21], we assumed that medicines produced and distributed illegally (e.g. “black market”, illegal internet sites) were substandard or FFC.

The results obtained in the sub-studies in Rome and Zagreb & Pula suggest that the tool is of added value in regions with a higher representation of specific risk groups, such as fitness club clients with an estimated prevalence of 5% users of non-prescribed medicines/health care products: 4 cases correctly classified in this setting equals 3% of the enrolled patients. It may be of significance that the offices of the general practitioners’ network (Rome) were located in the vicinity of fitness centres and military barracks.

The retrospective assessment of the medical records did not reveal harm by FFCm’s which was missed by the initial scoring (there were no “false negatives”). Several reasons may account for this observation:

- short observation time,
- prevalence of FFCm contamination in the study sample lower than assumed, perhaps due to selective use of brands or classes of medicines by the respective health care sites,
- large dose variability among individual units and batches of FFCm’s as demonstrated by Venhuis et al. (2013) [22] who concluded: “…In the worst case, counterfeit or unauthorised medicines are not recognised as such or health risk is not identified giving rise to ”false negative“ results…”.

## Conclusions

We consider this “decision aid” a systematic tool for physicians to find in medical practice patients harmed by FFCm’s. The tool can be integrated into routine patient questioning, diagnosis and treatment planning. The structure (questionnaire, watch-list, scoring forms) permits easy updates as new information becomes available. However, for being implemented in practice, scoring and searching the watch-list should be made more practical and less time-consuming. This would require digitalisation. The tool could be particularly useful in the ambulatory care setting in regions with a level of 10–20% contamination of the health care system by FFCm’s or in specific population groups at elevated risk of FFCm’s and health care products from uncontrolled outlets. Regular communication, systematic updates between the health authorities and the medical community are important to build and maintain a “pool” of expertise and knowledge and to foster motivation. Both aspects are part of the concept and operationalised tool. These principles are a cornerstone of a successful application of the tool, acceptance by physicians, and may contribute to find and reduce health damage caused by FFCm’s. Further development could focus on the digitalisation of the tool, the inclusion of suitable biomarkers of harm and on possible uses of the tool in training or as “aide memoire” to raise the awareness of physicians of FFCm’s and associated health damage.

## Additional files

Additional file 1: Questionnaire and scoring form of the tool (“Decision aid”). (DOCX 29 kb)

Additional file 2: Table S1. Frequency ranking: signs and symptoms, medical history, use of medicines and health care products. (DOCX 30 kb)

## Abbreviations

*EDQM*: European Directorate for the Quality of Medicines & HealthCare
*FFC*: Falsified, falsely labelled (counterfeit)
*Positive likelihood ratio (LR+) Negative likelihood ratio (LR-*): the ability of a test to discriminate between people likely to have a disorder and those less likely to have a disorder is determined by the test‘s likelihood ratio (LR). The ratio of true positives to false positives (LR+) is the likelihood ratio for a positive test result being correct. The ratio of false negatives to true negatives (LR-) is the likelihood ratio for a negative test result being correct.

## Footnotes

1 The EDQM coordinates the work programme carried out by the Council of Europe Committee of Experts on Minimising Public Health Risks posed by Counterfeiting of Medical Products and Similar Crimes (CD-P-CMED). The experts represent national authorities.

2 The national OMCLs are important partners of the competent authorities as regards the detection of FFCm’s on the markets and the analytical determination of quality defects. Networking among OMCLs in Europe is supported via the European Network of Official Control Laboratories coordinated by the EDQM [12].

3 The “Clopper/Pearson” Calculator [14] did not calculate confidence intervals for the likelihood ratios (LR+/LR-).
