# Supplementary material for: The development and appraisal of a tool designed to find patients harmed by falsely labelled, falsified (counterfeit) medicines
Source: BMC Health Serv Res. 2017 Jun 20;17:419. doi: 10.1186/s12913-017-2235-y (PMC5477164; doi:10.1186/s12913-017-2235-y)
Supplement: Supplementary file 1 — Questionnaire and scoring form of the tool (“Decision aid”). (DOCX 29 kb) [file 12913_2017_2235_MOESM1_ESM.docx]

**Additional File 1:**

Tool: Questionnaire and scoring form

**1. Questionnaire:**

**Patient information**

Year (Date) of birth:

Sex:

**Symptoms, medical history (known diseases), medicines and healthcare products‘ use**

1. 1. What symptoms made you seek medical help?

1.1 If you suffer from more than one symptom, which symptom is most important for you?

1.2 Which other symptoms do you experience?

1. 2. What diseases do you know that you actually suffer from (*medical history*)?
2. 3. What medicines do you use regularly?
3. 4. Do you use any other healthcare products like dietary supplements (such as vitamins, minerals, herbs)?

| Name of product | Dosage per day | How long? | Where are purchased? |
| --- | --- | --- | --- |
|  |  |  |  |
|  |  |  |  |

Who gave advice on the dosage and method of use?

| Coach |  | Internet |  |
| --- | --- | --- | --- |
| Friend |  | In the gym |  |
| Family member |  | Doctor |  |
| Dealer |  | Pharmacist |  |

5. Personal life-styles**:**

*List of appropriate life-style questions (exposure to falsely labelled, falsified (counterfeit) medicines and illegal health care products (FFCm’s):*

*(Annotation: Depending on the classes of medicines selected for use in the individual sub-study, either a complete extract (watch-list: life-style questions) was provided in item 5. or a version limited to the symptoms, life-styles and medicines or health care products relevant for the selection).*

**Antibiotic medicines**

- Did you recently take antibiotics to treat infections? Do you suffer from acne?
- Do you suffer from abdominal pain or dysmenorrhea?
- Do you take drugs to improve your brain function?
- Do you suffer from mycotic infections?
- Do you use any medication against allergic conditions?
- Do you suffer from vascular disease, stroke or myocardial infarction?

**Antiobesity**

- Do you suffer from constipation?
- Which products do you use to reduce weight? Do you want to lose weight?
- What are you doing to stay young and lean? Did you ever use medication for weight reduction?

**Related life-style medicines**

- Are you starting to suffer from starting baldness?
- Do you want to grow eye lashes?
- Do you want a darker skin without being in the sun (tan quickly)?
- Do you suffer from sleeplessness? Do you have other sleep disorders? Do you regularly travel in different time zones?

**Anabolics**

- Are you a strength athlete / body builder? Are you a professional athlete?
- Are you working out? What are you doing to enhance your athletic performance?

**Erectile dysfunction**

- Have you bought or used tablets/injections to enhance sexual performance/penile erection?
- How do you maintain/ improve your sex-life?
- Are you using libido enhancing herbs/ medicines?
- Are you experiencing loss of libido? How do you counter loss of libido caused by alcohol / obesity / diabetes / medication?
- Do you take any products to prolong your erection?
- Do you take any products to shorten the refractory time?

**Psycho-analeptics**

- Are you using drugs to stay awake and/or to enhance your concentration to study? Are you feeling nervous because of drugs that you use to study?
- Are you using the medication of someone else to enhance your concentration or for studying?
- Do you need any medications in order to being able to focus on your work?
- Do you need to stay awake for studying?

**2. Scoring form**

1. Could the most important/other symptoms (Reply to Question 1.1- 1.2) be reasonably associated to medical history (Reply to Question 2)?

☐ Yes ☐ No *(Yes = 0 points, No= 20 points)*

2. Are the most important symptoms (Reply to Question 1.1) included in the Watch-list: **”Leading symptoms”**?

☐Yes ☐ No *(Yes = 20 points, No= 0 points)*

3. Is the medicine and/or healthcare product used by the patient (Replies to Questions 3 and 4) included in the watch-list: Medicines frequently identified in Europe as falsely labelled, falsified (counterfeit) (FFCm’s) (comprising also illegal health care products)?

☐ Yes ☐ No *(Yes = 20 points, No= 0 points)*

4. Has the patient a life-style which suggests contact with FFCm’s or health care products (Replies to Questions 5-….)?

☐ Yes ☐ No *(Yes = 20 points, No= 0 points)*

5. Have the patients’ most important signs and symptoms been described in pharmacovigilance reports or scientific literature or other literature as symptoms caused by *FFCm’s* ?

☐ Yes ☐ No *(Yes = 20 points, No= 0 points)*

Please quote reference, if available:

Sum of scores for reply: (0- 20-40-60-80-100 points)

Harm caused by FFCm’s probable (Scores more or equal 40): ☐

Harm caused by FFCm’s unlikely (Scores less 40): ☐
