# Supplementary material for: The development and appraisal of a tool designed to find patients harmed by falsely labelled, falsified (counterfeit) medicines
Source: BMC Health Serv Res. 2017 Jun 20;17:419. doi: 10.1186/s12913-017-2235-y (PMC5477164; doi:10.1186/s12913-017-2235-y)
Supplement: Supplementary file 2 — Frequency ranking: signs and symptoms, medical history, use of medicines and health care products. (DOCX 30 kb) [file 12913_2017_2235_MOESM2_ESM.docx]

**Additional File 2**

Table S1 Frequency ranking: signs and symptoms, medical history, use of medicines and health care products.

| **Health care setting/ Selected classes of medicines** | **Signs &symptoms (%) (WHO ATC) (descending frequency)** | **Medical history (WHO ATC) (%) (descending frequency)** | **Medication (WHO ATC) (%) (used before consultation) (descending frequency)** | **Health care products (%) (descending frequency)** |
| --- | --- | --- | --- | --- |
| *Ambulatory* Yerevan (Armenia)/ Antibiotics J01) | \| 36 \| Respiratory system \| \| \| --- \| --- \| --- \| \| 22 \| Genito urinary system/sex hormonal \| \| \| 22 \| Dermatological \| \| \| 16 \| Alimentary tract/metabolic \| \| \| 8 \| Sensory organs \| | \| 34 \| Respiratory system \| \| --- \| --- \| \| 32 \| Alimentary tract/ metabolic \| \| 12 \| Genito-urinary system/ sex hormonal \| \| ..8 \| Dermatological \| | \| 18 \| Nasal preparations \| \| --- \| --- \| \| 16 \| Analgesics \| \| 12 \| Antibiotics/chemotherapeutics for dermatological use \| \| 10 \| Stomatological preparations \| \| 10 \| Antibacterials for systemic use \| | \| 18 \| \| Herbal preparations \| \| --- \| --- \| --- \| \| ..6 \| \| Herbals (Erectile dysfunction) \| \| ..6 \| \| Biologically active supplements (Sportsman hormone) \| \| ..6 \| Vitamins \| \| |
| *Ambulatory* Zagreb& Pula (Croatia)/ Anti-obesity Preparations, Anabolic Agents for Systemic Use, Diuretics, Sex Hormones & Modulators of the Genital System, Urologicals, Psycho-analeptics | \| 22.9 \| Musculo-skeletal system \| \| --- \| --- \| \| 20 \| Respiratory system \| \| 10 \| Nervous system \| \| 10 \| Alimentary tract /metabolic \| \| ..4.3 \| Sensory organs \| \| ..4.3 \| Cardiovascular system \| | \| 32.9 \| Cardiovascular system \| \| --- \| --- \| \| 17.1 \| Alimentary tract/metabolic \| \| 15.7 \| Musculo-skeletal system \| \| ..8.6 \| Endocrinological disorder (systemic hormonal system) \| | \| 17.1 \| Psycholeptics \| \| --- \| --- \| \| 17.1 \| Antihypertensives \| \| ..4.3 \| Drugs obstructive airway diseases \| \| ..4.3 \| Analgesics \| \| ..4.3 \| Anti-obesity preparations excl. diet products \| | \| 15.7 \| Vitamins \| \| --- \| --- \| \| 15.7 \| Supplements \| \| .4.3 \| Herbal preparations \| \| .4,3 \| Herbals (Erectile dysfunction) \| |

| Table S1 cont. Frequency ranking: signs and symptoms, medical history, use of medicines and health care products. | | | | |
| --- | --- | --- | --- | --- |
| **Health care setting/ Selected classes of medicines** | **Signs &symptoms (%) (WHO ATC) (descending frequency)** | **Medical history (WHO ATC) (%) (descending frequency)** | **Medication (WHO ATC) (%) (used before consultation) (descending frequency)** | **Health care products (%) (descending frequency)** |
| *Ambulatory* Rome (Italy)/  Anabolic Agents for Systemic Use, Diuretics, Sex Hormones and Modulators of the Genital System, Urologicals | \| 26.7 \| Nervous system \| \| --- \| --- \| \| 20 \| Respiratory system \| \| 10 \| Cardiovascular system \| \| 10 \| Musculo-skeletal system \| | \| ..8.3 \| Cardiovascular system \| \| --- \| --- \| \| ..8.3 \| Respiratory system \| \| ..8.3 \| Alimentary tract/metabolic \| | \| ..6.7 \| Antihypertensives \| \| --- \| --- \| \| ..6.7 \| Genito-urinary system/sex hormones \| \| ..1.7 \| Analgesics \| \| ..1.7 \| Alimentary tract/metabolic preparations \| | \| 20 \| Supplements \| \| --- \| --- \| \| 20 \| Vitamins \|   Including: Protein (amino acid) vitamin mix, creatin mix, “Thermoburner Animal stack” mix. |
| *In-patient* Aalst (Belgium)/ Anti-obesity Preparations, Anabolic Agents for Systemic Use, Diuretics, Sex Hormones and Modulators of the Genital System, Urologicals, Psycho-analeptics | \| 54.8 \| Musculo-skeletal system \| \| --- \| --- \| \| 11.9 \| Alimentary tract/metabolic \| \| 9.5 \| Genito-urinary system/sex hormonal \| \| ..7.1 \| Nervous system \| \| ..7.1 \| Cardiovascular system \| | \| 23.8 \| Cardiovascular system \| \| --- \| --- \| \| 19.0 \| Alimentary tract/metabolic \| \| ..9.5 \| Nervous system \| \| ..9.5 \| Respiratory system \| \| ..9.5 \| Musculo-skeletal system \| | \| 21.4 \| Antihypertensives \| \| --- \| --- \| \| 21.4 \| Analgesics \| \| 11.9 \| Psycholeptics \| \| ..9.5 \| Anti-infectives for systemic use \| \| ..9.5 \| Corticosteroids dermatological preparations \| | \| 16.7 \| Supplements \| \| --- \| --- \| \| .4.8 \| Vitamins \| \|  \|  \| |
| *In-patient* Reykjavík (Iceland)/  Anti-obesity Preparations | \| 26.8 \| Alimentary tract/metabolic \| \| --- \| --- \| \| 24.2 \| Musculo-skeletal system \| \| 18.8 \| Nervous system \| \| ..7.4 \| Respiratory system \| | \| 34.9 \| Alimentary tract/metabolic \| \| --- \| --- \| \| 21.5 \| Nervous system \| \| 19.5 \| Cardiovascular system \| \| 15.4 \| Musculo-skeletal system \| | \| 22.1 \| Antihypertensives \| \| --- \| --- \| \| 21.5 \| Psycholeptics \| \| 10.1 \| Alimentary tract / metabolic \| \| ..6.7 \| Thyroid therapy \| | \| 57.7 \| Vitamins & Supplements (Combi) \| \| --- \| --- \| \|  \|  \| |

Table S1 cont. Frequency ranking: signs and symptoms, medical history, use of medicines and health care products.

| **Health care setting/ Selected classes of medicines** | **Signs &symptoms (%) (WHO ATC) (descending frequency)** | **Medical history (WHO ATC) (%) (descending frequency)** | **Medication (WHO ATC) (%) (used before consultation) (descending frequency)** | **Health care products (%) (descending frequency)** |
| --- | --- | --- | --- | --- |
| **Total (%)** | \| 21.8 \| Musculo-skeletal system \| \| --- \| --- \| \| 16.2 \| Alimentary tract/metabolic \| \| 14.8 \| Respiratory system \| \| 14.6 \| Nervous system \| | \| 24.3 \| Alimentary system \| \| --- \| --- \| \| 18.1 \| Cardiovascular system \| \| 10.2 \| Musculo-skeletal system \| \| ..9.7 \| Nervous system \| | \| 13.2 \| Antihypertensives \| \| --- \| --- \| \| 13.2 \| Psycholeptics \| \| ..5.7 \| Analgesic \| \| ..4.3 \| Alimentary tract / metabolic \| | \| 23.2 \| Vitamins & Supplements (Combi) \| \| --- \| --- \| \| .8.1 \| Supplements \| \| .7.5 \| Vitamins \| \| .3.2 \| Herbal preparations \| \| .1.6 \| Herbals (Erectile dysfunction) \| |
